# Phantom validation of 4D flow: independent validation of vortex ring volume quantification using planar laser-induced fluorescence

**DOI:** 10.1186/1532-429X-17-S1-P38

**Published:** 2015-02-03

**Authors:** Johannes Töger, Sebastian L Bidhult, Johan Revstedt, Marcus Carlsson, Håkan Arheden, Einar Heiberg

**Affiliations:** 1 Cardiac MR group Lund, Dept. of Clinical Physiology, Lund University, Lund, Sweden; 2 Numerical Analysis, Centre for Mathematical Sciences, Lund University, Lund, Sweden; 3 Department of Biomedical Engineering, Faculty of Engineering, Lund University, Lund, Sweden; 4 Department of Energy Sciences, Lund University, Lund, Sweden

## Background

Previous studies suggest that vortex ring formation in the left ventricle of the human heart is a sensitive marker of cardiac diastolic function and overall cardiac health [1]. However, measurement of quantitative vortex ring parameters using 4D phase contrast magnetic resonance (4D PC-MR) has not previously been validated. Therefore, the purpose of this study was to validate measurement of vortex ring volume (VV) by 4D PC-MR using planar laser-induced fluorescence (PLIF) as the reference standard in a phantom setup.

## Methods

**Figure 1:**
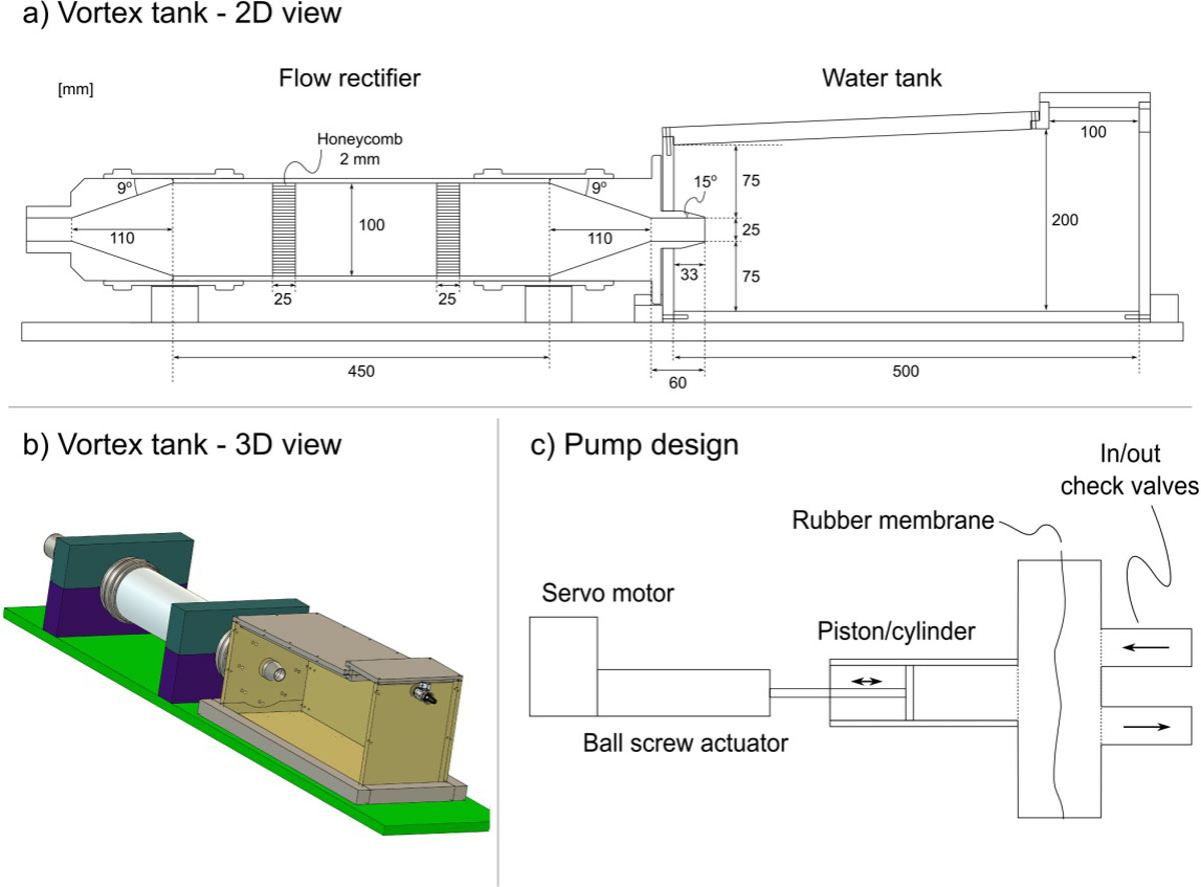
**Design and construction of the vortex ring flow phantom.** a) 2D view of the flow rectifier and vortex ring tank. All measures are in millimeters (mm). b) 3D view of the flow rectifier and vortex ring tank. c) Pump design. A servo motor powers a ball screw linear actuator, which in turn moves a piston-cylinder apparatus. A rubber membrane separates the flow medium from the pump to prevent abrasive particles from entering the piston/cylinder apparatus.

**Figure 2:**
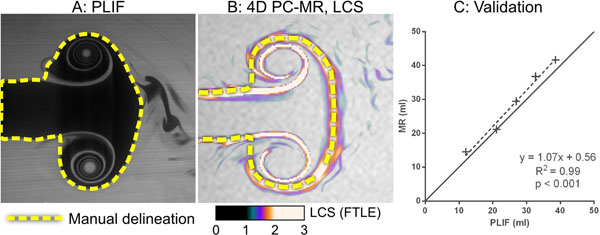
**Data analysis in PLIF and 4D PC-MR images and quantitative results for vortex ring volume (VV).** Panel A: Analysis of PLIF images. Panel B: Analysis of 4D PC-MR data using Lagrangian Coherent Structures (LCS). Panel C: Comparison of vortex ring volume (VV) at the five pump settings.

We constructed a pulsatile pump and a water tank with a 25mm nozzle (Figure 1), and five different pump settings with different pulse volumes and velocities were used. PLIF was performed using the fluorescent dye Rhodamine 590 (Rhodamine 6G, Exciton Inc., Ohio, USA) excited using a 532 nm Nd:YAG laser in a 1 mm vertical laser sheet. Images were acquired 400 ms after pump initiation, i.e. after complete vortex ring formation. Nozzle stroke volumes were measured using 2D PC-MR (voxel size 2.4x2.4x6 mm, VENC 50 cm/s). 4D PC-MR was acquired at 1.5T with 3x3x3 mm voxels and 50 ms temporal resolution. Vortex ring volume (VV) was quantified in PLIF by manual delineation (Figure 2A), and in 4D PC-MR data using manual delination of Lagrangian Coherent Structures (Figure 2B), a method for analysis of flow data that enables detection of the vortex ring boundary [2].

## Results

Stroke volumes ranged from 12-37 ml. Vortex ring volume (VV) showed excellent agreement between PLIF and 4D PC-MR (Figure 2C, R^2^ = 0.99, bias 2.4±1.5 ml).

## Conclusions

This study shows that vortex ring volume (VV) can be reliably quantified using 4D PC-MR.

## Funding

This study was supported by Swedish Research Council grants VR 621-2005-3129, VR 621-2008-2949, and VR K2009-65X-14599-07-3, National Visualization Program and Knowledge Foundation grant 2009-0080, the Medical Faculty at Lund University, Sweden, the Region of Scania, Sweden and the Swedish Heart-Lung Foundation.
